# Does maternal consumption of nutritive and non-nutritive sweeteners result in offspring hypertension?

**DOI:** 10.3389/fnut.2025.1464269

**Published:** 2025-01-22

**Authors:** You-Lin Tain, Chien-Ning Hsu

**Affiliations:** ^1^Division of Pediatric Nephrology, Kaohsiung Chang Gung Memorial Hospital, Kaohsiung, Taiwan; ^2^Institute for Translational Research in Biomedicine, Kaohsiung Chang Gung Memorial Hospital, Kaohsiung, Taiwan; ^3^College of Medicine, Chang Gung University, Taoyuan, Taiwan; ^4^Department of Pharmacy, Kaohsiung Chang Gung Memorial Hospital, Kaohsiung, Taiwan; ^5^School of Pharmacy, Kaohsiung Medical University, Kaohsiung, Taiwan

**Keywords:** developmental origins of health and disease, pregnancy, sweetener, hypertension, fructose, sugar, sugar-sweetened beverage

## Abstract

The consumption of nutritive and non-nutritive sweeteners (NNS) has increased significantly in recent decades. The nutritional status of pregnant women plays a crucial role in determining the likelihood of their offspring developing hypertension in adulthood. While NNSs provide a sweet taste without adding to sugar intake, emerging evidence suggests that maternal consumption of not only nutritive sweeteners (such as fructose) but also NNS may lead to adverse outcomes in offspring, including hypertension. This review provides an overview of the latest research connecting maternal intake of sweeteners to the long-term risk of hypertension in offspring. We examine proposed mechanisms underlying the programming of offspring hypertension by sweeteners, encompassing oxidative stress, dysregulated nutrient sensing signals, abnormal renin-angiotensin system, transcriptome changes, and dysbiotic gut microbiota. Additionally, we outline preventive strategies that can help alleviate offspring hypertension programmed by maternal diets high in sweeteners. Recent advancements in understanding the mechanisms through which maternal consumption of nutritive and non-nutritive sweeteners contributes to offspring hypertension offer promise for addressing this widespread health concern at its developmental roots. Nonetheless, further research is needed to educate the public about the safety of sweetener consumption during pregnancy and lactation.

## 1 Introduction

Hypertension is a widespread health challenge, affecting nearly half of the global adult population (1). Research involving both humans and animals suggests that hypertension may originate early in life (2–4), a concept known as the developmental origins of health and disease (DOHaD) hypothesis (5). An adverse in utero environment can cause morphological and functional changes during fetal development, increasing the likelihood of the offspring developing diseases later in life. Specifically, maternal imbalanced nutrition has been associated with early life insults.

The consumption of nutritive and non-nutritive sweeteners (NNS) has increased in recent decades (6, 7). Nutritive sweeteners add calories and affect blood sugar, whereas NNS are low-calorie or calorie-free. A significant source of added sugars in the diet is sugar-sweetened beverages (SSBs) (6). Emerging evidence suggests a positive association between the intake of SSBs and the global rise in obesity and cardiometabolic disorders (7). Consequently, the World Health Organization advices reducing sugar intake. Of note is that pregnant women consume as much or even more sweeteners than the general population (8, 9). Although the consumption of sweeteners during gestation may negatively impact gestational results and the health of the next generation, debates continue, and the mechanisms remain unclear (9).

**Figure 1:**
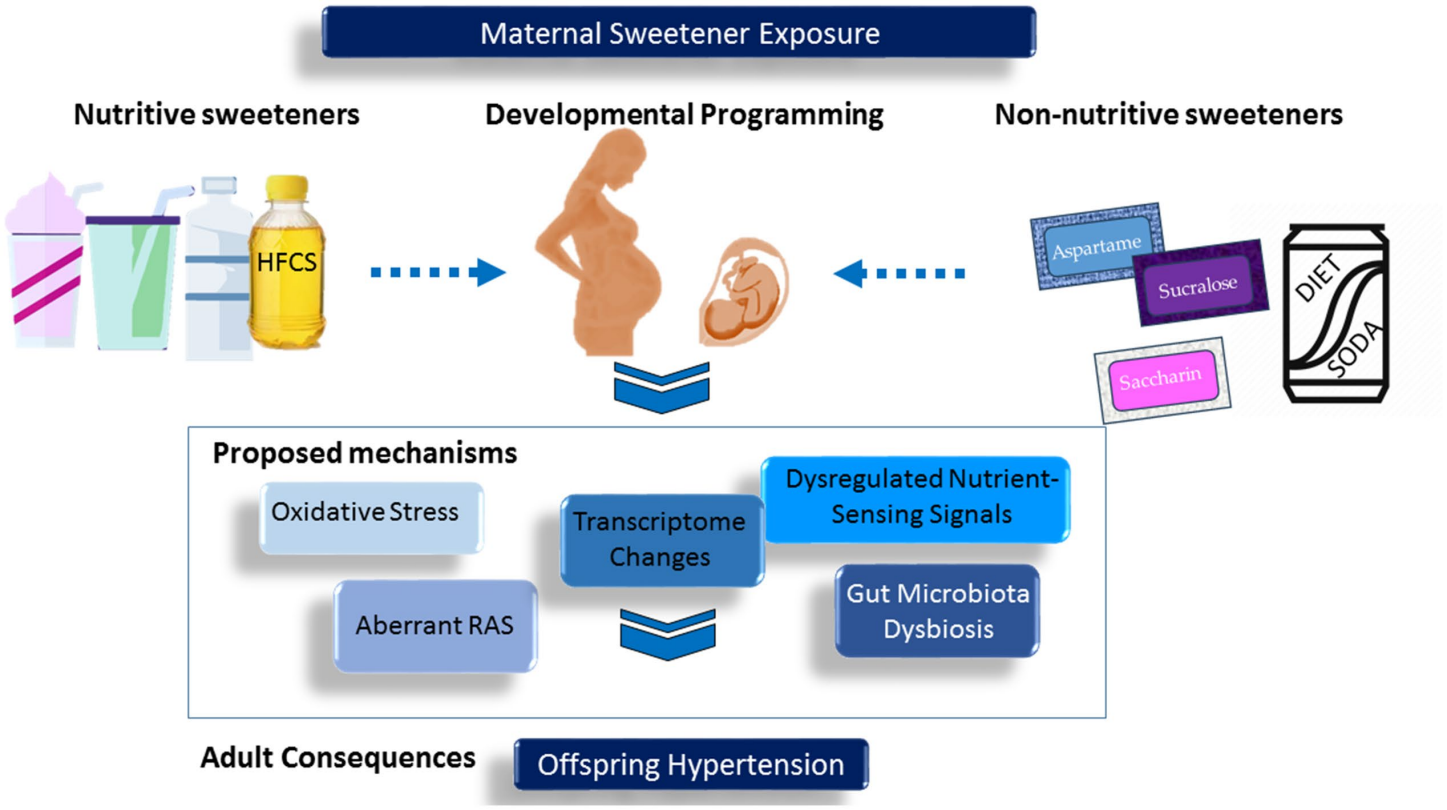
Schematic representation of the interrelationships between maternal exposure to sweeteners, developmental programming, and offspring hypertension.

The most common nutritive sweeteners are sucrose (i.e., table sugar) and high-fructose corn syrup (HFCS). Artificially sweetened beverages, also known as NNB, are also a significant source of dietary sugar. A comprehensive meta-analysis involving 35 studies revealed a positive correlation between both SSBs and artificially sweetened beverages (ASBs) with hypertension (10). Nevertheless, there is still uncertainty surrounding the impact of nutritive and non-nutritive sweeteners consumption during pregnancy on the blood pressure (BP) of offspring. In this review, we will examine and consolidate existing literature concerning both nutritive and non-nutritive sweeteners. We will explore evidence linking maternal exposure to sweeteners with offspring hypertension and discuss proposed mechanisms. For a comprehensive visual representation, please refer to Figure 1.

## 2 Exposure to nutritive and non-nutritive sweeteners during pregnancy and lactation

### 2.1 Nutritive sweeteners

Nutritive sweeteners are substances that provide calories and are commonly used to sweeten foods and beverages. They encompass both natural and processed sugars. Natural sugars include sucrose, fructose, glucose, lactose, maltose, honey, maple syrup, sugar alcohols, molasses, etc. Processed sugars, on the other hand, are sweeteners that have been extracted and refined from their natural sources. Well-known examples of processed sugars include table sugar (sucrose), brown sugar, and HFCS.

Nutritive sweeteners are extensively used not only for their sweetness but also for their ability to improve the preservation and texture of foods. However, excessive consumption of these sweeteners can result in a myriad of health concerns, including diabetes, obesity, and dental cavities. A meta-analysis of human studies demonstrated a positive association between fructose consumption and elevated BP, as well as certain components of metabolic syndrome (11). Additionally, in a cohort study investigating the intake of SSBs and ASBs during gestation, an elevated likelihood of preterm delivery was observed (12). Although fructose consumption may have harmful effects on the developing fetus, no long-term studies have yet evaluated the influence of maternal fructose consumption on the risk of hypertension in offspring (13). The health implications for human offspring resulting from high fructose consumption during gestation remain largely unclear.

### 2.2 Non-nutritive sweeteners

Over the past decade, corresponding with trends in the general population is the increase in consumption of NSS during pregnancy (8). For pregnant and lactating women, as well as the general public, seven non-nutritive sweeteners are currently approved for use in foods and consumption: aspartame, acesulfame-K, saccharin, sucralose, neotame, stevia, and advantame (9, 14). Their structures and relative sweetness, as well as their Acceptable Daily Intake (ADI), are summarized in Table 1. Among these, acesulfame-K and aspartame are the most extensively utilized in foods, with childbearing women and their children being the primary consumers (15, 16). Considering that a variety of NNSs are commonly combined in a single food item, accurately estimating the actual intake of NNS in pregnant women poses a challenge due to their widespread presence in numerous foods, beverages, supplements, and medications (15, 16).

**Table 1:** Available non-nutritive sweeteners (NNSs).

NNS	Molecular formula	Relative sweetness	ADI (mg/kg of body weight)
Aspartame	C_14_H_18_N_2_O_5_	200x	50
Acesulfame-K	C_4_H_4_KNO_4_S	200x	15
Saccharin	C_7_H_5_NO_3_S	300x	15
Sucralose	C_12_H_22_O_11_	600x	5
Neotame	C_20_H_30_N_2_O_5_	7,000–13,000x	0.3
Stevia	C_38_H_60_O_18_	100-300x	4
Advantame	C_24_H_30_N_2_O_7_	20,000x	32.8

While certain NNSs are completely metabolized (e.g., aspartame), the majority (e.g., acesulfame K, saccharin, and sucralose) circulate in the body without being metabolized. Accordingly, transference of NNS to a fetus via the placenta and to an infant through breast milk is plausible. Animal research revealed that acesulfame K, saccharin, and sucralose can be found in the urine of fetus or amniotic fluids, indicating potential transmission via cord blood in utero (17, 18). Though human observational studies have suggested an association between NNS consumption and adulthood metabolic diseases (14), what remains unexplored is the impact of maternal exposure to NNS on the risk of offspring hypertension, as no conclusion has been drawn in this area of research.

## 3 The link between sweeteners and blood pressure

Excessive nutritive sweetener consumption is significantly linked to hypertension, primarily due to metabolic effects that promote obesity, insulin resistance, and elevated uric acid levels. NNSs may serve as beneficial substitutes for reducing sugar intake, though their effects on BP can vary.

Both sucrose and HFCS contain fructose, which has been implicated in increasing BP through various mechanisms. Sucrose enzymatically breaks down into one fructose molecule and one glucose molecule, while HFCS contains free fructose and glucose in varying ratios. HFCS-55, a prevalent variety of HFCS utilized in sweetening beverages, contains a composition of 55% fructose, 42% glucose, and 3% oligosaccharides (19). In the Western diet, the main contributors of dietary fructose are sucrose (commonly known as table sugar) and HFCS, despite the natural occurrence of fructose in fruits.

Due to its nearly complete first-pass extraction by the liver, where it is quickly converted to fructose 1-phosphate, fructose metabolism differs significantly from glucose metabolism (20). Unlike glucose, which is transported by various glucose transporters, the transport of fructose occurs via the facilitative glucose transporter 5, utilizing a specific and passive mechanism. The role of glucose transporters in hypertension, however, re-mains largely unknown. Animal studies have shown that the mechanisms by which excess fructose increases BP include increased salt absorption, elevated uric acid levels, chronic stimulation of the sympathetic nervous system, and endothelial dysfunction (21). Utilizing RNA next-generation sequencing (NGS) technology, the transcriptome expression in the offspring was studied within a maternal high-fructose diet model (22, 23). Notably, the onset of hypertension in offspring was related to several differentially expressed genes (DEGs) in the kidneys of neonate offspring, particularly those involved in insulin signaling, fatty acid metabolism, fructose metabolism, gluconeogenesis, and glycolysis (23).

Incorporating 372 studies, a previous review revealed that there is no conclusive evidence regarding the positive or detrimental effects of NNSs on BP (24). The three primary artificial sweeteners used in soft drinks and diet sodas are aspartame, stevia, and sucralose. An acute injection of aspartame was shown to reduce systolic BP in spontaneously hypertensive rats (25), though its long-term effects remain unclear. Stevia may cause slight reductions in BP, but the magnitude of these effects is minimal (26). Sucralose, which has a molecular structure nearly identical to table sugar, has little impact on BP. In healthy older adults, intra-duodenal administration of sucralose was not related to any BP changes (27). Compared to a high fructose-fed rat model, saccharin was found to elevate fasting blood glucose levels without affecting BP (28). The associations between BP and other alternative sweeteners remain unclear. Overall, adopting a balanced diet with reduced added sugars and mindful use of alternative sweeteners can help improve BP management.

## 4 Maternal sweetener exposure-induced offspring hypertension

While numerous studies have evaluated the effects of nutritive and non-nutritive sweeteners on BP, there is limited human research on how maternal exposure to these substances impacts fetal development and disease risk in offspring, particularly concerning hypertension. Animal studies have shown that fructose or sucrose alone can alter fetal programming (23). However, most studies have examined these sugars as components of a Western diet that also includes fat and salt.

Studies summarized in Table 2 indicate that maternal consumption of sweeteners during gestation and lactation induces hypertension in adult offspring (29–43). Most studies focused on fructose or sucrose, with none examining non-nutritive sweeteners. The commonly used species in these studies are rats and mice. Excess fructose was administered either in chow (60%) or drinking water (10–40%). Similarly, sucrose was administered either in chow (26%) or in 20% drinking water. Considering that each month of rodent life roughly corresponds to three human years (44), Table 2 presents the age at measurement from 8 to 52 weeks, equating to human ages from adolescence to middle adulthood. Considering the multifactorial nature of hypertension, several studies have investigated the combined impact of maternal fructose intake and other pivotal elements of a Western diet, such as high levels of salt and fat consumption. These studies have demonstrated that these elements together synergistically elevate BP in adult offspring (45–48).

**Table 2:** Maternal consumption of sweeteners programs blood pressure in rodent models.

Types of sweetener intake	Periods Pregnancy/Lactation	Strain/Sex	Programming mechanisms	Age at measure (weeks)	Ref.
Fructose					
60% HF diet	Yes/Yes	Male SD rats	Transcriptomic changes in the kidneys and increased sEH protein and activity	12	(29)
60% HF diet	Yes/Yes	Male SD rats	Transcriptomic changes in the kidneys, dysregulated EDHF and arachidonic acid metabolism	12	(30)
60% HF diet	Yes/Yes	Male SD rats	Dysregulated arachidonic acid pathway and enhanced eEH	12	(31)
60% HF diet	Yes/Yes	Male and female SD rats	Aberrant renin-angiotensin system	12	(32)
60% HF diet	Yes/Yes	Male SD rats	Gut microbiota dysbiosis	12	(33)
60% HF diet	Yes/Yes	Male SD rats	Reduced SCFA levels and increased TMAO levels	12	(34)
60% HF diet	Yes/Yes	Male SD rats	Increased oxidative stress and AT1R expression in the brain	12	(35)
60% HF diet	Yes/Yes	Male SD rats	Sympathetic activation, increased ROS production, and T-lymphocyte activation	24	(36)
10% *w*/*v* fructose in drinking water	Yes/Yes	Female SD rats	Decreased eNOS expression	17	(37)
13% or 40% *w*/*v* fructose in drinking water	Yes/Yes	Male SD rats	Transcriptomic changes in the hypothelamus, increased AT1R, and increased TLR4	8	(38)
20% *w*/*v* fructose in drinking water	Yes/Yes	Male and female C57BL/6 J mice	Aberrant RAS	32	(39)
20% *w*/*v* fructose in drinking water	Yes/Yes	Male and female C57BL/6 J mice	Increased the expression of PRR, NKCC2, NHE3, and NCC	32	(40)
10% *w*/*v* fructose in drinking water	Yes/Yes	Male C57BL/6 J mice	Not evaluated	52	(41)
Sucrose					
20% w/v sucrose in drinking water	Yes/No	Male SD rats	Increased AT1R	13	(42)
Sucrose-rich diet (26% of total energy)	Yes/Yes	Male and female C57BL/6 J mice	Increased sympathetic efferent tone and renal noradrenaline content	12	(43)

Most studies have focused on fructose. Although various doses and durations of fructose consumption have been used to induce hypertension in offspring, no study has explored the dose-or duration-dependent effects of maternal fructose intake on offspring BP, an area that requires further investigation. While sucrose (which contains both fructose and glucose) and fructose share some metabolic pathways, glucose and fructose are metabolized differently in the body. Glucose is used by most cells for immediate energy, whereas fructose is primarily processed in the liver, where it can be converted into glucose or fat. Given these differences in metabolism, further research is needed to determine whether hypertension in offspring induced by maternal sucrose or fructose intake results from the same mechanisms or if they differ.

Various molecular pathways have been identified as contributing to the development of hypertension in offspring as a result of maternal diets rich in sweeteners (2–4). These pathways include oxidative stress, abnormalities in the renin-angiotensin system (RAS), disturbances in nutrient sensing signals, alterations in the transcriptome, and dysbiosis of gut microbiota. Each of these mechanisms will be examined extensively.

### 4.1 Oxidative stress

Oxidative stress is crucial in fetal programming due to the fetus’s heightened sensitivity to oxidative damage, attributed to its low antioxidant capacity (49). The generation of reactive oxygen species (ROS) mediated by added sugars contributes to the development of hypertension (50). Research has shown that offspring exposed to a maternal diet high in fructose exhibit hypertension, accompanied by elevated levels of oxidative stress (35, 36). In rat offspring exposed to maternal high-fructose intake, elevated ROS levels were observed, along with increased expression of the pg91phox subunit of NADPH oxidase and decreased levels of superoxide dismutase 2 in the brain (35). Another study revealed that a maternal high-fructose diet caused oxidative stress in tissues and increased the expression of pro-inflammatory cytokines in the spleen of adult offspring (36). Additionally, oxidative stress may reduce the production of nitric oxide (NO) by elevating the synthesis of asymmetric dimethylarginine (ADMA), which acts as an endogenous inhibitor of NO synthase (51). Melatonin, an antioxidant, has been shown to avert hypertension in offspring programmed by a maternal high-fructose diet by reducing plasma ADMA levels and enhancing NO production (29).

It’s important to note that even when consumed within recommended safe limits, aspartame and its metabolites have the potential to disturb the balance between oxidants and antioxidants, leading to oxidative stress-related damage (52). Likewise, several artificial sweeteners like saccharin, acesulfame-K, sucralose, and stevia have been associated with adverse health conditions related to oxidative stress (53–55). Therefore, whether oxidative stress induced by maternal consumption of artificial sweeteners contributes to hypertension in offspring requires further investigation.

### 4.2 Aberrant RAS

Comprising various angiotensin peptides with a range of biological functions, the RAS is intricately associated with hypertension (56, 57). The RAS cascade begins with renin, which is derived from its precursor, prorenin (58). The interaction between circulating renin and prorenin with their (pro) renin receptor (PRR) triggers angiotensin II (ANG II)-independent signaling cascades, leading to local ANG II production. Typically, activation of the classic axis—angiotensin-converting enzyme (ACE)/ANG II/ANG II type 1 receptor (AT1R)—promotes vasoconstriction, oxidative stress, and inflammation, all of which contribute to hypertension (57). Hypertension observed in offspring as a result of maternal high-fructose diet programming correlates with abnormal activation of the classic RAS axis, evidenced by increased levels of PRR, angiotensinogen, ACE, and AT1R in organs that regulate BP, such as the kidneys, blood vessels, and brain (32, 35, 38–40, 42).

One study found that a maternal high-fructose diet could activate the RAS across multiple generations (39). Significant increases in BP were observed in the first-and second-generation offspring when compared to the control group. However, this phenomenon did not manifest in the third and fourth generations. Intriguingly, exhibited by the third-generation offspring were the highest levels of serum ANG II, renin and aldosterone. Furthermore, the fructose diet led to elevated mRNA expression of RAS-related genes in the kidneys from the first to the third generation of rat offspring (40). For instance, maternal high-fructose intake induces offspring hypertension coincided with increased expression of several sodium transporters, including Na/K/2Cl cotransporter (NKCC2), sodium/hydrogen exchanger 3 (NHE3), and Na + -Cl − cotransporter (NCC) (40). However, other studies have reported inconsistent findings regarding the impact of maternal fructose consumption on sodium transporters (45).

Conversely, early blockade of the classic RAS has garnered attention as a reprogramming strategy to avert hypertension caused by various maternal insults (59). Revealed in one study is the potential for preventing hypertension in adults whose mothers consumed a high-fructose diet by administering the renin inhibitor aliskiren to offspring aged 2–4 weeks (32). Despite significant advancements in the availability of different RAS-based interventions, there is still a need for deeper investigation into their reprogramming effects on maternal sugar intake-induced hypertension in offspring. Notably, a metabolite of aspartame has been found to inhibit ACE (60). However, whether maternal consumption of aspartame and other artificial sweeteners has beneficial or harmful effects on offspring BP remains to be clarified.

### 4.3 Dysregulated nutrient-sensing signals

Throughout pregnancy, mechanisms for sensing nutrients detect specific nutritional components to ensure the proper coordination of fetal development and organ function (61). Maternal dietary nutrients serve as metabolic substrates for various processes through the mediation of nutrient-sensing signals. Disrupted nutrient-sensing signals during gestation can lead to adverse fetal programming, contributing to the development of hypertension originating from developmental issues (62). Several key signals involved in programmed hypertension include AMP-activated protein kinase (AMPK), peroxisome proliferator-activated receptors (PPARs), PPARγ coactivator-1α (PGC-1α), and silent in-formation regulator transcript (SIRT) (63).

AMPK is activated by a decline in cellular energy status and serves as a glucose sensor (64, 65). AMPK and SIRT1 mediate the phosphorylation and deacetylation of PGC-1α, respectively (65). The downstream signaling of PGC-1α impacts PPARγ, which regulates the expression of particular PPAR target genes involved in hypertension with developmental origins (66, 67).

In a rat model where both maternal and post-weaning diets were high in fructose, there was a observed decrease in the renal mRNA expression of PGC-1α, AMPK, and PPARs (68). Conversely, resveratrol, which acts as an AMPK activator, can modulate these nutrient-sensing pathways to activate PPAR target genes, thereby offering protection against hypertension in offspring (69). Additionally, maternal prebiotic therapy, which has been shown to prevent elevated BP in adult offspring of fructose-fed dams, was connected to increased protein abundance of phosphorylated AMPKα2 (33). These findings suggest a potential connection between maternal sugar intake and dysregulated nutrient-sensing signals underlying hypertension with developmental origins.

### 4.4 Transcriptome changes

Maternal high-fructose intake can induce long-term transcriptome changes (22, 69), which is a key player involved in hypertension of developmental origins (23, 70, 71). Notably, different organs respond uniquely to developmental programming, leading to organ-specific alterations in the transcriptome (69).

Our previous research revealed significant transcriptome changes in the 1-day-old male offspring kidneys exposed to maternal high-fructose diet (22). In total, 2,706 DEGs were identified, comprising 1,214 upregulated and 1,492 downregulated genes. Among them, 20 out of 2,706 high-fructose diet-related DEGs are associated with regulation of BP. Notably, we also identified 14 significantly regulated Kyoto Encyclopedia of Genes and Genomes (KEGG) pathways. Notably, the PPAR signaling pathway emerged as a nutrient-sensing signaling mechanism linking maternal fructose intake to offspring hypertension. Furthermore, our findings implicate *Hpgds*, *Cyp2c23*, *Ptges*, and *Ptgds*, which are involved in arachidonic acid metabolism, in the development of hypertension in offspring induced by a maternal high-fructose diet.

Arachidonic acid metabolites involved in the pathogenesis of hypertension (72). The metabolism of arachidonic acid can lead to the formation of epoxyeicosatrienoic acids (EETs) through the action of cytochrome P450 (CYP) epoxygenases. Following their formation, EETs undergo conversion into dihydroxyeicosatrienoic acids (DHETs) through the action of soluble epoxide hydrolase (SEH) (73). EETs have vasodilatory effects, where-as DHETs exert vasoconstrictive actions. Maternal high-fructose diet can enhance SEH activity and renal DHET in the kidneys of 3-week-old male rat offspring (30). Conversely, our follow-up study revealed that targeting on arachidonic acid pathway by 15-Deoxy-Δ12,14-prostagandin J2 (15dPGJ2) or SEH inhibitor 12-(3-adamantan-1-yl-ureido)-dodecanoic acid prevented offspring hypertension pro-grammed by maternal high-fructose intake (31).

### 4.5 Gut microbiota dysbiosis

The gut microbiota forms a symbiotic community of trillions of microbes from more than 1,000 species, playing an active role in modulating the host’s physiological functions (74), including the regulation of BP (75–77). Interactions between the host and microbes are primarily facilitated by microbial-derived metabolites. Among these, dietary-derived bacterial metabolites, such as trimethylamine N-oxide (TMAO), tryptophan derivatives, short-chain fatty acids (SCFAs), and branched-chain amino acids, are notably involved in regulating BP homeostasis (75–77).

Small amounts of dietary fructose are effectively metabolized by the gut, but higher doses can exceed the intestinal capacity, resulting in alterations in gut microbiota composition and dysbiosis (78, 79). The effects of alternative sweeteners on gut microbiota have also been documented (80). Among NNSs, consumption of saccharin (81, 82), sucralose (83), and acesulfame K (84, 85) has been shown to alter gut microbiota composition. Although studies do not consistently identify specific changes in taxa in response to different NNSs (86), a consistent finding is the depletion of *Akkermansia muciniphila* when exposed to NNSs like saccharin, sucralose, and acesulfame K in both adult and infant mice (87, 88). Additionally, aspartame can be rapidly metabolized and related to SCFA production, especially propionate production (17).

Offspring hypertension induced by maternal fructose intake is correlated with dysbiosis of gut microbiota (89). Supplementing the drinking water of pregnant rats with 10% fructose led to significant alterations in the maternal microbiome, particularly diminishing beneficial microbes like *Lactobacillus* and *Bacteroides* (90). Likewise, a maternal diet high in fructose also influenced the microbiome of rat offspring. In adult male offspring, hypertension programmed by maternal consumption of high fructose was associated with a reduced presence of the genus *Akkermansia* (34). In another study, adult rat progeny born to dams who were fed a diet comprising 60% fructose throughout gestation and lactation periods exhibited an elevation in the *Firmicutes* to *Bacteroidetes* ratio (68), a microbial marker of hypertension.

Conversely, treatments such as prebiotics, postbiotics, probiotics, and modulation of microbial metabolites have shown potential in mitigating hypertension induced by maternal fructose diets (85). For instance, perinatal supplementation with *Lactobacillus casei* prevented hypertension in adult offspring born to dams received excessive intake of fructose (33). Additionally, perinatal supplementation of inulin, a widely recognized prebiotic, conferred protection against maternal high-fructose diet-primed hypertension (33). The beneficial effects of inulin are linked to elevated plasma propionate levels and the reversal of reduced expression of SCFA receptors caused by a high-fructose diet. Postbiotics, encompassing diverse constituents like SCFAs, have also demonstrated potential in reversing hypertension programming (91). Acetate, a SCFA, can regulate BP via its receptors (77). In a rodent model of maternal high-fructose diet, supplementing acetate during the perinatal period showed beneficial effects against offspring hypertension (34). Moreover, adjusting the microbial metabolite TMAO has shown efficacy in shielding against hypertension induced by fructose in offspring. TMAO, derived from trimethylamine (TMA), has been linked to the risk of cardiovascular disease (92, 93). Inhibition of microbe-dependent TMAO and TMA formation, achieved using the choline analog DMB (94), protected adult rat offspring from hypertension programmed by a maternal diet high in fructose (34). Despite the documented issues in offspring’s gut microbiota associated with the maternal consumption of fructose, the programming effects of alternative sweeteners on offspring BP has yet to be fully understood.

### 4.6 Others

Given that sweeteners are only one component of the maternal diet and various maternal nutritional factors can contribute to offspring hypertension, there may be other mechanisms, aside from those mentioned above, that play a role in the pathogenesis of nutritional programming associated with hypertension of developmental origins (95–97). One potential mechanism is epigenetic regulation, a key factor in developmental programming and hypertension (98–100). Changes in gene expression could result from epigenetic mechanisms, including aberrant DNA methylation, histone modification, and microRNAs (miRNAs) (101). One study found that maternal high-fructose diet-induced hypertension in multigenerational offspring is linked to an enrichment of active histone marks, such as H3Ac and H3K4me2, and a decrease in repressive histone marks, such as H3K9me3 and H3K27me3, on the pro(renin) receptor promoter (40). While the exact effects of epigenetic mechanisms related to maternal sweetener intake and their influence on the risk of hypertension in offspring are not fully understood, they warrant further investigation. Additionally, NSSs can interact with taste receptors and the taste-selective G-protein, *α*-gustducin, which are involved in body weight maintenance and the risk of chronic diseases, including hypertension (102, 103). Therefore, the potential role of taste receptor signaling should be considered, despite the current lack of supporting information.

## 5 Preventive strategies for sweetener-related offspring hypertension

Primary prevention aims to avert hypertension before it manifests. To this end, it is crucial to avoid excessive consumption of sweeteners during pregnancy and lactation. Additionally, early-life interventions can reprogram the molecular mechanisms underlying sweetener-related hypertension with developmental origins, thereby preventing the onset of hypertension in adulthood (4).

First, several antioxidants have been investigated for their potential to counteract the adverse effects of excessive fructose consumption (104). These include L-arginine (105), L-citrulline (106), L-taurine (107), folic acid (108), melatonin (109), N-acetylcysteine (110), *α*-lipoic acid (111), polyphenols (112), and resveratrol (113). However, only a limited number of interventions have been investigated in the context of maternal sweetener intake-induced hypertension in offspring. Melatonin, a multifunctional hormone primarily secreted by the pineal gland, possesses strong antioxidant properties (114, 115). In a rat model of a maternal high-fructose diet, perinatal melatonin treatment was shown to decrease ADMA levels and increase NO bioavailability (29). Melinjo seed extract is rich in polyphenols, including resveratrol (116). Numerous studies have demonstrated that dietary polyphenol intake is associated with positive health outcomes, including reduced hypertension (117). One study found that maternal intake of melinjo seed extract during lactation mitigated the increase in BP induced by high-fructose consumption in female adult offspring (37). Another study indicated that maternal resveratrol supplementation reduced renal oxidative stress and prevented programmed hypertension induced by high-fructose intake (68).

Secondly, several interventions targeting the RAS have demonstrated benefits in protecting against hypertension with developmental origins. These interventions include renin inhibitors (32), ACE inhibitors (118), angiotensin receptor blockers (ARBs) (119), and AT1R antisense (120). Of these, only the aliskirin (a renin inhibitor) and losartan (an ARB) have been specifically evaluated for its reprogramming effects against hypertension programmed by maternal high-fructose diets in offspring (32, 35).

Thirdly, targeting nutrient-sensing pathways such as AMPK and PPAR has shown potential in reprogramming hypertension with developmental origins in several animal models (66, 67). Metformin, known for its ability to lower blood glucose by targeting gluconeogenesis and activating AMPK (121), has been particularly effective. Oral administration of metformin to young offspring exposed to a maternal high-fructose diet prevented programmed hypertension, coinciding with a decrease in AT1R and increases in SIRT1 and PGC-1α (35). Additionally, perinatal use of 15dPGJ2, a PPARγ ligand, has been shown to avert hypertension programmed by maternal high-fructose intake in offspring (31).

Lastly, gut microbiota-targeted therapies show promise in preventing developmental programming induced by maternal fructose consumption (89). Among these interventions are prebiotics, probiotics, and postbiotics. While fecal microbiota transplantation has demonstrated the ability to alleviate metabolic syndrome-related traits in a rat model on a high-fructose diet (122), its potential effects on the offspring of fructose-fed dams remain unexplored While numerous probiotic microbes, prebiotics, and postbiotics have been linked to health benefits (123–125), there is currently a lack of robust evidence supporting their roles in sugars and alternative sweeteners-induced developmental programming.

Only one study has documented that perinatal supplementation with *Lactobacillus casei* (a probiotic) or inulin (a prebiotic) protects adult male rat progeny against hypertension programmed by a maternal diet high in fructose (33). Acetate, a SCFA acting as post-biotics, also displayed benefits against maternal high fructose diet-primed offspring hypertension (34). Another approach, involving TMA inhibition through DMB treatment throughout gestation and lactation periods, also protects adult offspring from hypertension complicated by a maternal diet high in fructose (34). Understanding the reprogramming effects of functional foods containing prebiotic-like components, such as polyphenols and resveratrol, on developmental hypertension induced by maternal sugar intake will be critical for their potential clinical application.

Although most animal studies have focused on fructose-induced programmed hypertension, several mechanisms have been hypothesized to be responsible for the programming effects of NSSs (126, 127). These include shaping gut microbiota (80), activation of intestinal sweet taste receptors (128), induction of oxidative stress (129), and changes of brain neurotransmitters (130). Whether these mechanisms can also be targeted for reprogramming deserves further investigation.

Most studies on fructose-induced programmed hypertension focus on males, leaving the role of sex differences—proposed in other models of programmed hypertension (4, 131–133)—unclear. The renal transcriptome in hypertension is sex-specific in offspring programmed by a maternal high-fructose diet (32), which may offer a potential protective mechanism for females. However, whether sex differences influence the programming effects of NSSs and whether they are beneficial or harmful to fetal development remains uncertain. A better understanding of sex-dependent mechanisms underlying sweetener-induced offspring hypertension could lead to novel sex-specific prevention strategies. This review offers a comprehensive overview of different interventions in early life that show promise in addressing hypertension related to maternal sugar intake with developmental origins. In spite of significant progress in animal research, the clinical translation of these findings remains a considerable challenge.

## 6 Conclusions and future directions

Emerging evidence from animal studies highlights the dangers of maternal sweetener consumption and elucidates the molecular mechanisms that program hypertension in adult offspring. Current research has provided insights into how early-life therapies targeting specific mechanisms can reprogram maternal sweetener-induced hypertension in offspring. These reprogramming approaches include antioxidants, RAS-based interventions, AMPK activators, and gut microbiota-targeted therapies. Given that sweeteners can program multiple organs, resulting in various phenotypes in adult offspring, there is a pressing need for more studies focusing on organ-specific reprogramming effects. Although the primary focus of this review is the maternal consumption of sweeteners, the potential paternal nutritional epigenetic effect cannot be entirely excluded (119), despite the lack of supporting information.

Many NNSs pass through the placenta and are detected in amniotic fluid and breast milk, indicating a potential risk for the developmental programming of adult diseases (134). Although NNSs provide sweetness without contributing to sugar intake, animal models suggest that maternal consumption of NNSs may program offspring to develop obesity or metabolic syndrome (134–136), which are known risk factors for hypertension. However, scant data exist regarding the effect of maternal intake of NNS on offspring BP in both human and animal research. As maternal intake of NNSs continues to rise, it becomes imperative to assess their long-term safety and their effects on offspring.
